# Special Issue “Molecular Scale Design, Synthesis, and Application of Macroporous, Mesoporous, and Microporous Materials”

**DOI:** 10.3390/ijms27062592

**Published:** 2026-03-12

**Authors:** María Teresa Colomer

**Affiliations:** Instituto de Cerámica y Vidrio (ICV), CSIC, c/Kelsen 5, E-28049 Madrid, Spain; tcolomer@icv.csic.es; Tel.: +34-91-735-5840; Fax: +34-91-735-5843

The aim of this Special Issue is to emphasize the significance of molecular-scale design, synthesis, and application of macro-, meso-, and microporous materials in order to achieve optimal performance across diverse fields. These materials are commonly classified according to their pore diameters. Based on the criteria established by the IUPAC, microporous materials possess pore diameters smaller than 2 nm, mesoporous materials range from 2 to 50 nm, and macroporous materials exhibit diameters larger than 50 nm [1]. It is well known that porous materials have numerous current and emerging applications in various areas. They are widely used for gas separation and storage, liquid phase adsorption and catalysis in the fields of food processing, biotechnology, pharmaceuticals, petrochemicals, water remediation, etc. In addition, they are also employed in production and energy storage. When their development is controlled at the molecular level, it becomes possible to tailor their internal structure and introduce specific chemical functionalization, thereby optimizing performance for targeted applications. Recent research in this area focused on the use of in situ techniques and modeling to further understand the dependence of the functional properties on the molecular assembly and pore formation. The fundamental mechanisms at the molecular level, directing self-assembly and pore evolution under varying conditions, especially for hierarchical and hybrid systems, are still not fully understood. In this sense, multidisciplinary characterization such as real-time microscopy combined with in situ structural and spectroscopic techniques offer a deeper insight than before into the mechanisms of pore formation and structure–property relationships. Furthermore, molecular simulations are necessary for accelerating the development of tailored porous materials. In this respect, integrated computational–experimental protocols and machine learning can predict porous architectures prior to synthesis. However, these types of studies are still limited [2]. In addition, advanced design and novel synthesis strategies are necessary in order to develop hierarchical and multiscale pore structures with improved mass transport and multifunctionality within a single architecture [3,4]. Low yields, template removal, use of hazardous solvents, or synthesis routes that require high energy consumption currently limit the production of materials with promising applications. For these reasons, it is important to develop scalable and environmentally friendly synthesis routes at low temperatures, in which templates are biodegradable or recyclable. Template-directed assembly and bottom-up techniques allow a precise control of pore sizes and distributions. In addition, chemical etching [5] and micelle-based assembly [6] are tools for shaping the pore structures during synthesis. These processes can generate hierarchical porosity and dynamic self-healing porous networks that keep structural integrity, minimizing or eliminating solvent use [7,8]. Those novel routes will allow the preparation of controlled pore architectures, achieving atomic-level control over pore geometry, connectivity, and surface functionality while maintaining scalability and structural robustness. Advances in inverse design strategies will enable researchers to tailor pore size distributions, surface chemistry, and hierarchical architectures for a specific field such as selective catalysis, gas separation, energy storage, and molecular sensing. Different types of porous materials in diverse application fields are currently receiving special attention ([9], and references there in), such as functionalized mesoporous silica nanoparticles [10,11], metal–organic frameworks (MOFs) [12,13], porous organic polymers [14], covalent organic frameworks (COFs) [15], carbon-based materials [16,17,18,19,20], etc., and in future they will be studied more. Functional integration is also an important issue. Embedding catalytic, photonic, electronic or magnetic properties directly into porous scaffolds is a strategy that will continue to be optimized in the future [21,22]. Overall, molecular control of porosity will enable the development of more selective, efficient, and sustainable materials, fostering technological progress in these areas, especially for energy storage and conversion, advanced separation, catalysis under real conditions, health care, etc. This Special Issue presents representative studies illustrating these advances, including a molecular dynamics simulation investigation connecting atomistic modeling with membrane performance in practical applications.

Conclusively, the future of the research of macro-, meso- and microporous materials will be determined by data-driven design, precisely controlled synthesis combined with in situ reaction monitoring and advanced characterization techniques, and resource- and energy-efficient green manufacturing.
